# Impact of the COVID-19 Pandemic on Primary Care Referral Patterns and Resource Utilization in a Hospital Emergency Department: A Comparative Pre- and Later-Pandemic Study

**DOI:** 10.3390/medsci14030407

**Published:** 2026-07-21

**Authors:** Angel Iván Díaz-Salado, Francisco Javier García-Sánchez, Alicia Fuente-Gaforio, Andrés Estropá-Zapater, Irene Pérez-Arévalo, Sandra Moreno-Ruiz, María Teresa Sánchez-Álvarez, Natalia Mudarra-García

**Affiliations:** 1 Emergency Room Service, Hospital Universitario Doce de Octubre, Instituto de Investigación Sanitaria Hospital 12 de Octubre (IMAS12), 28041 Madrid, Spain; angelivan.diaz@salud.madrid.org; 2 Emergency Room Service, Hospital Universitario Infanta Cristina, Instituto de Investigación Sanitaria Hospital Puerta de Hierro Segovia Arana (IDIPHISA), 28981 Madrid, Spain; andres.estropa@salud.madrid.org (A.E.-Z.); iparevalo@salud.madrid.org (I.P.-A.); smruiz@salud.madrid.org (S.M.-R.); 3 Medical Department, Faculty of Medicine, University Complutense of Madrid, 28040 Madrid, Spain; 4 Hospital Universitario Ramón y Cajal (IRYCIS), 28034 Madrid, Spain; alicia.fuente@salud.madrid.org (A.F.-G.); natalia.mudarragarcia@ceu.es (N.M.-G.); 5 Data Management Unit, Hospital Universitario Infanta Cristina, Instituto de Investigación Sanitaria Hospital Puerta de Hierro Segovia Arana (IDIPHISA), 28981 Madrid, Spain; mteresa.sancheza@salud.madrid.org; 6 Nursing Department, University San Pablo-CEU, CEU Universities, Urbanization Montepríncipe, 28660 Boadilla del Monte, Spain

**Keywords:** COVID-19, pandemic, emergency department, primary care, referral patterns, healthcare utilization, health services research

## Abstract

Background: The COVID-19 pandemic profoundly disrupted healthcare utilization patterns at both primary care (PC) and hospital emergency department (ED) levels. This study aimed to assess the impact of the pandemic on referral patterns from PC to a hospital ED and on the resource consumption associated with those referrals. Methods: A descriptive, retrospective, longitudinal comparative study with multivariable sensitivity analyses was conducted at a first-level hospital of Madrid (Spain). All consecutive PC-to-ED referrals received during two observation windows were included: a pre-pandemic period (1 June–31 December 2019; n=946) and a Later-Pandemic period (1 January–30 June 2022; n=1797). Sociodemographic characteristics, referral form quality, diagnostic specialty, and in-ED resource utilization variables were collected and compared using $\chi^{2}$, Student’s *t*-test, and Mann–Whitney U tests as appropriate. To assess seasonal confounding, multivariable logistic and ordinal regression models adjusting for calendar season, age, and sex were performed for the primary resource-utilization outcomes. Results: A total of 2743 referrals were analyzed. The monthly referral rate increased by approximately 122% between periods (135/month vs. 300/month). A statistically significant but clinically negligible difference in mean age was observed (53.1±18.3 vs. 54.9±19.0 years; p=0.015); no significant sex differences were found. Referral form completion improved significantly for clinical history (94.5% vs. 98.2%; p<0.001). Orthopedics referrals nearly tripled (5.8% vs. 18.4%), while respiratory/COVID-19-related referrals represented 22.0% of the 2022 caseload. ED length of stay between 3 and 6 h increased from 13.0% to 42.8% (p<0.001), while the need for urgent blood tests fell from 68.9% to 56.0% (p<0.001), hospital admission from 68.4% to 10.9% (p<0.001), and referral to another center from 12.3% to 0.9% (p<0.001). In multivariable sensitivity analyses, the period effect remained statistically significant after adjustment for calendar season, age, and sex for hospital admission (adjusted OR =0.062; 95% CI: 0.037–0.106; p<0.001) and urgent blood test utilization (adjusted OR =0.470; 95% CI: 0.326–0.678; p<0.001), although study period and season remain partly confounded by the non-overlapping design. Conclusions: During the later-pandemic period, PC-to-ED referrals increased substantially while being associated with fewer complementary investigations and fewer hospital admissions. This study documents between-period differences in referral patterns and ED resource utilization rather than causal effects; the differences persisted after adjustment for seasonality but cannot be definitively attributed to the pandemic. Future multi-center longitudinal studies are needed to confirm these trends and clarify their underlying mechanisms.

## 1. Introduction

The Spanish National Health System operates through a multilevel, multidisciplinary structure in which primary care (PC) and hospital emergency departments (EDs) constitute the two main entry points for patients [1]. Both levels of care have experienced a sustained increase in demand over recent decades, making effective coordination between them essential for delivering safe, high-quality, and efficient care [2,3].

PC physicians have a pivotal role in regulating patient flow to EDs. Appropriate referral not only ensures that patients receive the level of care they require but also prevents unnecessary overcrowding of EDs, which is associated with increased adverse outcomes and healthcare costs [4,5]. The proportion of patients who may be safely managed at the PC level depends on the accessibility and technical capacity of PC services. This capacity includes the availability of point-of-care diagnostics and specialist teleconsultation [5,6]. When PC capacity is insufficient, patients who could be managed in the community end up attending the ED with benign or low-acuity conditions, straining hospital resources [4].

The COVID-19 pandemic, declared by the World Health Organization in March 2020, caused unprecedented disruption to health systems worldwide [7,8]. In Spain, as in many other countries, the successive pandemic waves forced a rapid reorganization of healthcare delivery at all levels: telehealth consultations were scaled up, elective activities were canceled, and infection control measures were implemented throughout the care pathway. Several studies have documented a sharp initial reduction in ED attendances during the first COVID-19 wave [9,10], followed by a gradual recovery and, in some centers, a rebound in demand during subsequent waves in 2021–2022.

However, the specific effect of the pandemic on PC-to-ED referral behavior, in terms of both volume and clinical complexity, remains poorly characterized, particularly for the post-confinement recovery period. Existing Spanish literature has primarily focused on within-hospital or within-ED impact [10], without explicitly examining the interface between PC and secondary care. Understanding how the pandemic reshaped this interface is crucial for informing future resource planning and identifying whether pandemic-era improvements in communication and clinical resolution may be sustained.

The study hospital is a first-level referral university hospital affiliated with a Spanish public university, serving a catchment area of approximately 200,000 inhabitants through eight main PC centers in its reference health district. In 2019, the ED received approximately 150 patients per day, of whom an estimated 7–8% were referred from PC. This setting, therefore, represents a suitable single-center model for studying the PC-ED interface.

Throughout this manuscript, the 2022 observation window is referred to as the “later-pandemic” period. This terminology is used descriptively and does not imply that the pandemic had ended: the World Health Organization declared the end of the Public Health Emergency of International Concern only in May 2023, and SARS-CoV-2 continued to circulate actively throughout the 2022 study period.

The objectives of this study were: (1) to characterize and compare the volume and clinical characteristics of PC-to-ED referrals between the pre-pandemic and Later-Pandemic periods; (2) to evaluate the quality of referral documentation; (3) to compare in-ED resource utilization and patient outcomes between the two periods; and (4) to assess whether calendar season confounded the observed between the differences of period through multivariable sensitivity analyzes.

## 2. Materials and Methods

### 2.1. Study Design and Setting

A descriptive, retrospective, longitudinal comparative study with multivariable sensitivity analyzes was conducted at the Emergency Department of a first-level referral university hospital serving a mixed urban-rural area in the southern metropolitan region of Madrid, Spain, affiliated with a national public university.

### 2.2. Study Periods and Population

**Figure 1:**
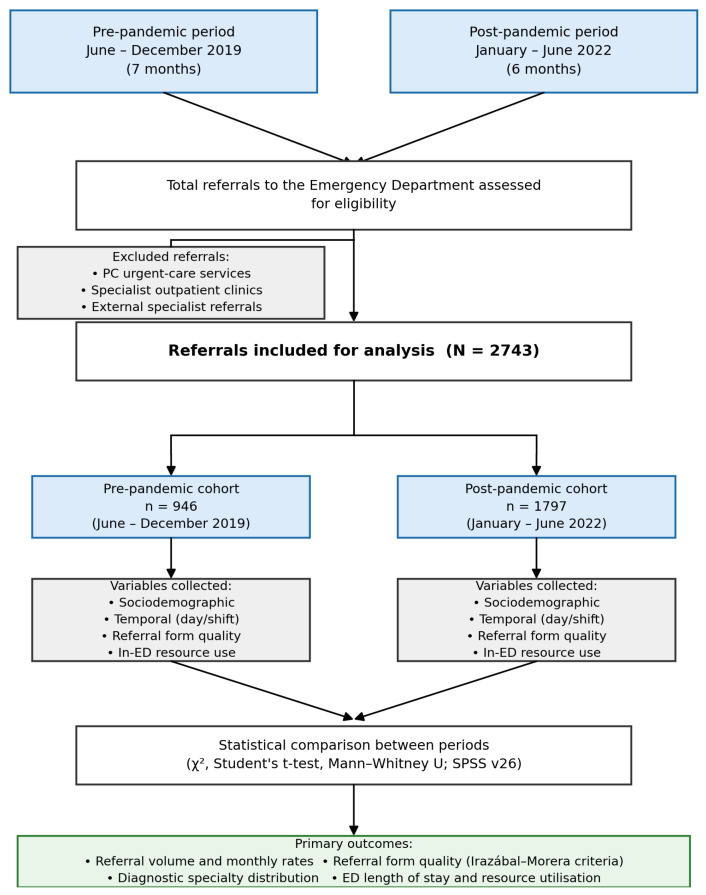
Flowchart of the study population. PC: primary care; ED: emergency department.

Two non-overlapping observation windows were selected (Figure 1):

- **Pre-pandemic period**: 1 June–31 December 2019 (7 months), preceding the onset of the COVID-19 pandemic in Spain.
- **Later-Pandemic period**: 1 January–30 June 2022 (6 months), after the main pandemic waves had subsided but while the healthcare system was still operating under the influence of pandemic-related structural changes.

All consecutive referrals from PC centers to the ED received during regular working hours (Monday to Friday, 08:00–21:00; morning shift 08:00–15:00 and afternoon shift 15:00–21:00) were included. Referrals originating from PC urgent-care services, specialist outpatient clinics, or external specialists were excluded.

### 2.3. Variables

The following variables were collected from anonymized referral forms and the electronic medical record:

**Sociodemographic variables:** age (years, continuous); sex (male/female); PC center of origin (eight centers, identified as PC Centers 1–8 in order of referral volume).

**Temporal variables:** day of the week (Monday to Friday); work shift (morning or afternoon).

**Referral form quality variables,** assessed using the validated quality criteria of Irazábal and Gutiérrez [11] as modified by Morera et al. [12]: presence of clinical history (yes/no); physical examination findings (yes/no); diagnostic impression or clinical judgment (yes/no); and reason for referral (specialist assessment; request for complementary tests; request for admission; appointment request).

**Clinical specialty:** diagnoses classified into Cardiology, Hematology, Infectious Diseases, Dermatology, Neurology, Gastroenterology, Pulmonology (including COVID-19), Orthopedics, ENT (Otorhinolaryngology), Nephrology, Urology, Gynecology, Ophthalmology, Psychiatry, General Surgery, Endocrinology, Rheumatology, Maxillofacial Surgery, Allergology, and Vascular Surgery.

**In-ED resource utilization and outcome variables:** need for urgent blood tests (yes/no); plain radiography (yes/no); advanced imaging (urgent ultrasound or CT scan) (yes/no); urgent specialist consultation (yes/no); intravenous treatment or plaster cast (yes/no); ED length of stay (<3 h; 3–6 h; 6–12 h; >12 h); need for ED observation (yes/no); hospital admission (yes/no); and referral to another center (yes/no).

### 2.4. Data Collection and Ethics

All data were extracted from a password-protected database specifically designed for this study and accessible only to the principal investigators. The study was approved by the Ethics and Research Committee of Hospital Universitario Puerta de Hierro (Approval No. PI 151/22, dated 18 July 2022) and conducted in accordance with the principles of the Declaration of Helsinki, the Spanish Data Protection Act (BOE-A-2018) and the European General Data Protection Regulation (EU 2016/679).

### 2.5. Statistical Analysis

Categorical variables were described as frequencies and percentages. Continuous variables were expressed as mean ± standard deviation (SD). Between-group comparisons used the $\chi^{2}$ test for categorical variables, Student’s *t*-test for normally distributed continuous variables, and the Mann-Whitney U test for non-normally distributed variables; ANOVA and Kruskal-Wallis tests were applied for comparisons involving more than two groups. Statistical significance was set at p<0.05. All analyzes were performed with IBM SPSS Statistics version 26.0 (IBM Corp., Armonk, NY, USA). To assess whether calendar season confounded the observed between-period differences in primary resource-utilization outcomes, multivariable sensitivity analyzes were performed. Binary logistic regression models were constructed for hospital admission and for urgent blood tests, incorporating study period (pre- vs. Later-Pandemic), calendar season (winter: January–February; spring: March–May; summer: June–August; autumn: September–November), age (years, continuous), and sex as covariates. Model performance was evaluated using the Nagelkerke pseudo-$R^{2}$ coefficient and the percentage of correctly classified cases. An ordinal logistic regression model with identical covariates was applied for ED length of stay (four ordered categories: <3 h, 3–6 h, 6–12 h, >12 h). Winter was used as the seasonal reference category in all models.

## 3. Results

### 3.1. Referral Volume and Sociodemographic Characteristics

A total of 2743 PC-to-ED referrals were analyzed: 946 (34.5%) in the pre-pandemic period (June–December 2019) and 1797 (65.5%) in the Later-Pandemic period (January–June 2022). Adjusting for the different observation lengths (7 vs. 6 months), the monthly referral rate increased from approximately 135 to 300 referrals per month, a 122% increase.

The mean age of patients was similar between periods (53.1±18.3 years in 2019 vs. 54.9±19.0 years in 2022; p=0.015), with no clinically meaningful difference. No significant sex differences were observed: 431 males (45.6%) and 515 females (54.4%) in 2019 vs. 829 males (46.1%) and 968 females (53.9%) in 2022 (p=0.775).

Referral distribution by day of the week showed significant differences (p=0.026); Wednesday was consistently the day with the highest number of referrals in both periods (223, 23.6% in 2019; 401, 22.3% in 2022). No significant shift differences were observed (p=0.283). Referral volume varied significantly by PC center (p=0.001): PC Centre 1 generated the most referrals in both periods (255 in 2019; 428 in 2022), followed by PC Centre 2 (180; 214), PC Centre 3 (159; 278), and PC Centre 4 (158; 350). Full data are presented in Table 1.

### 3.2. Referral Form Quality

Completion of the clinical history section improved significantly (94.5% in 2019 vs. 98.2% in 2022; p<0.001). The presence of a diagnostic impression also increased significantly (44.7% vs. 50.1%; p=0.008). No significant change was found for the physical examination section (81.2% vs. 83.7%; p=0.097).The most frequent reason for referral was specialist assessment in both periods (92.8% vs. 92.9%; p=0.282). Data are summarized in Table 2.

### 3.3. Diagnostic Specialty Distribution

The distribution of referrals by specialty changed significantly (p<0.001; Table 3). The most notable shifts were:

- **Orthopedics**: increased from 5.8% to 18.4%, nearly tripling, consistent with a surge in physical activity after the end of confinement.
- **Pulmonology + COVID-19**: 12.9% in 2019, rising to 22.0% in 2022 when COVID-19-specific referrals (7.7%) are combined with pulmonology (14.3%).
- **Gastroenterology**: decreased from 17.2% to 9.1%.
- **Hematology**: decreased from 7.1% to 2.7%.
- **Infectious Diseases**: decreased markedly from 5.7% to 0.5%.
- **Ophthalmology**: increased from 3.0% to 6.1%.
- **Vascular Surgery**: appeared as a new category in 2022 (1.1%).

### 3.4. Emergency Department Length of Stay

The distribution of ED length of stay changed substantially (p<0.001; Table 4). Stays of less than 3 h decreased from 67.9% to 32.7%, while stays of 3–6 h increased from 13.0% to 42.8%. Stays of 6–12 h increased from 1.9% to 10.9%, and stays exceeding 12 h decreased slightly from 17.2% to 13.6%.

### 3.5. In-ED Resource Utilisation and Outcomes

Compared with the pre-pandemic period, significant decreases were observed across multiple resource utilization variables (Table 4):

- **Urgent blood tests**: 68.9% vs. 56.0%; p<0.001.
- **Advanced imaging** (ultrasound or CT): 18.3% vs. 15.2%; p=0.037.
- **Intravenous treatment or plaster cast**: 44.3% vs. 31.9%; p<0.001.
- **ED observation**: 12.7% vs. 8.7%; p<0.001.
- **Hospital admission**: 68.4% vs. 10.9%; p<0.001.
- **Referral to another center**: 12.3% vs. 0.9%; p<0.001.

**Figure 2:**
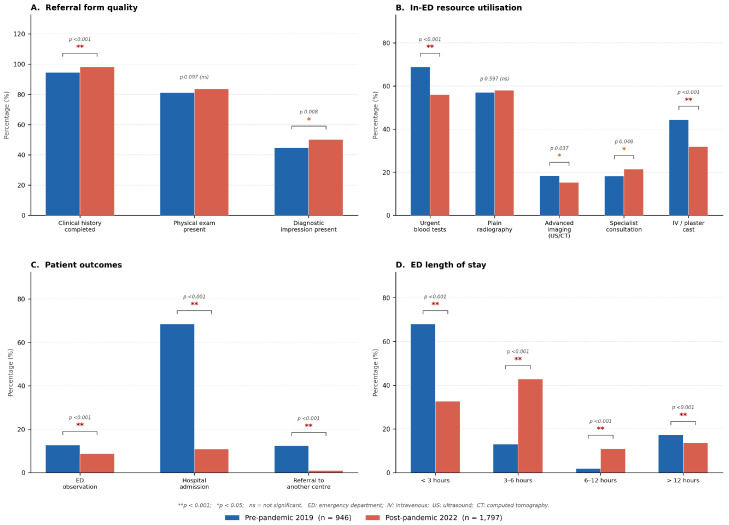
Comparison of key indicators between the pre-pandemic (2019) and Later-Pandemic (2022) periods. (**A**) Referral form quality; (**B**) in-ED resource utilisation; (**C**) patient outcomes; (**D**) ED length of stay distribution. Data are expressed as percentages. * p<0.05; ** p<0.001.

Conversely, urgent specialist consultation increased (18.2% vs. 21.4%; p=0.048), and plain radiography use remained stable (57.0% vs. 58.0%; p=0.597). Key indicators are visually summarized in Figure 2.

### 3.6. Sensitivity Analysis for Seasonal Confounding

Given the non-overlapping calendar months of the two observation windows (June–December 2019 and January–June 2022), multivariable regression analyzes were performed to determine whether calendar season confounded the primary between-period differences. Results for the binary logistic regression models are presented in Table 5.

For hospital admission, the global model was highly significant ($\chi^{2}=1094.2$; p<0.001; Nagelkerke $R^{2}=0.464$; correct classification 82.1%). After adjusting for season, age, and sex, the study period remained the dominant predictor, with an adjusted OR of 0.062 (95% CI: 0.037–0.106; p<0.001), representing a 94% reduction in the odds of hospital admission in 2022 relative to 2019, independent of season. Age was also independently associated with the outcome (OR =0.975 per year; 95% CI: 0.969–0.980; p<0.001). Calendar season was not globally significant as a predictor; however, a marginal association was observed specifically for autumn (p=0.012), a season concentrated in the pre-pandemic window, which warrants acknowledgment as a residual confounding element. Sex was not significantly associated with hospital admission (OR =1.022; 95% CI: 0.830–1.259; p=0.836).

For urgent blood tests, the model was significant ($\chi^{2}=142.2$; p<0.001; Nagelkerke $R^{2}=0.068$; correct classification 62.6%), with the study period remaining independently associated after seasonal adjustment (OR =0.470; 95% CI: 0.326–0.678; p<0.001). Calendar season was not statistically significant either at the global level (p=0.166) or for any individual seasonal category. Sex was not significant (OR =0.992; 95% CI: 0.847–1.161; p=0.918).

For ED length of stay, an ordinal logistic regression model ($\chi^{2}=312.1$; p<0.001; Nagelkerke $R^{2}=0.118$) confirmed a significant period effect (p<0.001) after controlling for season, age, and sex. Calendar season was not a significant predictor for any category (p>0.05 throughout), nor was sex (p>0.05). Older age was independently associated with longer ED stays (p<0.001).

Across all three models, study period was the dominant and statistically significant predictor of the observed outcome differences, while calendar season did not reach statistical significance. These findings indicate that seasonal variation does not materially confound the primary results of this study.

## 4. Discussion

This study provides a comprehensive comparison of PC-to-ED referral patterns and in-ED resource utilization between the pre-pandemic (2019) and Later-Pandemic (2022) periods at a first-level referral university hospital.

The principal finding is a substantial increase in referral volume during the later-pandemic period, accompanied by a reduction in in-ED resource consumption and hospital admission rates. In multivariable sensitivity analyses, the period effect on the primary outcomes remained statistically significant after adjustment for calendar season, age, and sex; however, because the two observation windows share no overlapping calendar months, study period and season remain partly confounded by design, and seasonality cannot be considered definitively excluded. The retrospective single-center design precludes causal inference. These findings are therefore best interpreted as documenting between-period differences in the observable characteristics of PC-to-ED referrals, rather than as demonstrated causal effects of the pandemic. Whether these differences reflect improved inter-level coordination, more selective referral, shifts in case mix, system-level reorganization, or reduced access to care are plausible but unproven explanations that cannot be distinguished with the available data.

### 4.1. Increase in Referral Volume

The monthly referral rate approximately doubled between the two study periods. This contrasts with the sharp initial decline in ED attendances documented during the first COVID-19 wave, attributed to patient avoidance of healthcare facilities, lockdown restrictions, and the repurposing of hospitals for COVID-19 management [9,10]. Our post-confinement data from 2022 capture a different dynamic: the pent-up demand accumulated during the pandemic, combined with a sustained rise in baseline PC caseload and the addition of COVID-19-related referrals, resulted in a substantially higher referral volume. Similar rebounds in emergency activity during 2021–2022 have been described by other Spanish authors [10,13].

The near-tripling of traumatology referrals (5.8% to 18.4%) is particularly noteworthy. This finding aligns with the widely reported surge in physical and sports-related injuries following the lifting of movement restrictions in 2021 [9], and may also partly reflect patients with accumulated musculoskeletal pathology that went unattended during the pandemic. By contrast, the sharp fall in infectious disease referrals (5.7% to 0.5%) likely reflects both improved telemedicine management of infectious episodes at the PC level and the diversion of COVID-19 cases to dedicated care pathways.

An additional factor potentially contributing to healthcare demand during the 2022 period is post-COVID-19 condition (long COVID), which may have generated referrals for persistent respiratory, cardiological, or neurological symptoms following acute infection. Our dataset did not capture prior SARS-CoV-2 infection status or post-acute sequelae, and we were therefore unable to quantify their specific contribution to referral volume or case mix; this represents an avenue for future research.

### 4.2. Improvement in Referral Form Quality

The improvement in clinical history completion (94.5% to 98.2%; p<0.001) and diagnostic impression documentation (44.7% to 50.1%; p=0.008) is a clinically relevant finding. High-quality referral documentation reduces iatrogenic errors, facilitates ED triage, and enables more efficient clinical assessment [14]. It is plausible that the pandemic-driven shift towards structured electronic referral platforms and telemedicine contributed to this improvement. However, the absence of a diagnostic impression in almost half of the referrals in 2022 (49.9%) remains a meaningful gap, and targeted training programs for PC physicians may be warranted. The validated quality framework of Irazábal and Gutiérrez [11] as modified by Morera et al. [12] provided a robust instrument for this assessment.

### 4.3. Changes in ED Resource Utilization

The dramatic fall in hospital admission rates from 68.4% in 2019 to 10.9% in 2022 (p<0.001) is the most striking finding of this study. Although the pre-pandemic figure may partly reflect complex or semi-urgent cases that were channeled through the ED, the Later-Pandemic figure suggests that a greater proportion of 2022 referrals were of lower acuity or were resolved definitively within the ED. The concurrent increase in ED stays of 3–6 h (13.0% to 42.8%) is consistent with this interpretation: patients required greater observation time within the ED but were ultimately discharged. Critically, multivariable sensitivity analyzes confirmed that this fall in hospital admission rates persisted robustly after adjusting for calendar season (adjusted OR =0.062; 95% CI: 0.037–0.106; p<0.001; Table 5), substantially reducing the plausibility of seasonal confounding as an alternative explanation. Nevertheless, this finding must be interpreted with considerable caution. Critically, we did not have access to institutional data on whether hospital admission criteria, ED clinical pathways, observation-unit practices, inpatient bed availability, or patient admission-qualification procedures changed between 2019 and 2022. Any of these organizational factors could have contributed substantially to the observed reduction, independently of any change in referral appropriateness or patient acuity. In the absence of such data, the magnitude of the admission decline cannot be attributed to improved clinical resolution, and the available data do not permit determination of whether all non-admitted patients received clinically appropriate care. This finding should therefore be regarded as hypothesis-generating and requiring confirmation in studies with access to institutional and case-mix data.

The reductions in urgent blood test requests (68.9% to 56.0%) and advanced imaging use (18.3% to 15.2%) likely reflect both a shift in case mix towards more musculoskeletal and respiratory presentations that require fewer laboratory investigations and an enhanced clinical resolution capacity within the PC setting, where general practitioners may have gained confidence in managing borderline cases through their pandemic experience. The increased use of telephone consultations between PC and hospital specialists during the pandemic [6] may also have enabled a more appropriate pre-referral work-up.

The increase in urgent specialist consultations within the ED (18.2% to 21.4%; p=0.048) may reflect a more selective referral process, with PC physicians retaining simpler cases and referring only those genuinely requiring specialist assessment, thereby increasing the average acuity of the referred population.

### 4.4. Temporal Patterns

The consistent predominance of Wednesday as the peak referral day in both periods aligns with previously reported patterns [2] and may reflect the accumulation of clinical decisions across the first half of the working week. The absence of significant differences by work shift suggests that PC-to-ED referral activity is relatively evenly distributed across morning and afternoon hours, which has implications for ED staffing planning.

### 4.5. Strengths and Limitations

Strengths of this study include the large consecutive sample from a real-world secondary care setting, the coverage of two epidemiologically distinct periods, and the use of validated quality criteria for referral form assessment. The retrospective design enabled complete data capture without participant drop-out.

Limitations include the single-center design, which restricts generalizability to other health districts with different demographic compositions or healthcare organizational models. The observation windows differ in duration (7 vs. 6 months), which was addressed by converting raw counts to monthly rates. Unmeasured confounders such as changes in GP workforce, adoption of new telemedicine platforms, or shifts in the catchment population during the pandemic may have contributed to the observed differences and cannot be disentangled from the pandemic effect itself.

The retrospective design precludes causal inference. Outcomes beyond the ED encounter were not available. Importantly, the multivariable models adjusted only for study period, calendar season, age, and sex; they did not account for other potentially influential confounders, including diagnostic case mix, referring PC centre, patient acuity or severity, institutional care pathways, inpatient bed availability, or changes in ED and hospital admission procedures between the two periods. Residual confounding from these unmeasured factors cannot be excluded and may account for part of the observed between-period differences.. Additionally, multiple endpoints were tested without a formal correction strategy; borderline *p*-values (notably p=0.037 and p=0.048) should therefore be interpreted with caution.

No validated patient acuity or severity scores were applied, so the observed reduction in resource utilization cannot be unambiguously attributed to improved referral appropriateness rather than to a lower acuity case mix or access constraints. Finally, while the term “Later-Pandemic” is used throughout for consistency with existing literature, the WHO declared only the end of the Public Health Emergency of International Concern in May 2023, not the end of the pandemic itself; the 2022 study period therefore occurred while SARS-CoV-2 continued to circulate actively.

A further important limitation concerns the non-equivalence of the two comparison windows. The pre-pandemic period spans June to December 2019, whereas the Later-Pandemic period spans January to June 2022; these intervals share no overlapping calendar months, which means that seasonal variation in referral demand cannot be excluded as a contributor to the observed differences.

While the multivariable models adjusted for calendar season, the fundamental two-period design cannot fully distinguish pandemic-related changes from secular trends, system reorganizations, or residual seasonal effects.

An interrupted time-series or segmented regression analysis using monthly data across multiple years, or a matched-month sensitivity analysis, would be required to attribute the observed differences specifically to pandemic-related effects. This study should therefore be interpreted as descriptive and hypothesis-generating rather than as evidence of causal impact.

## 5. Conclusions

Following the initial waves of the COVID-19 pandemic, PC-to-ED referrals increased substantially in volume, with a monthly rate approximately twice that of the pre-pandemic period. Despite this increase, referred patients required fewer complementary investigations, were significantly less frequently hospitalized, and were rarely transferred to other centers, while the quality of referral forms improved significantly.

In multivariable sensitivity analyses, these between-period differences remained statistically significant after adjustment for calendar season, age, and sex, with study period the dominant predictor across all primary outcomes; nonetheless, because the observation windows do not overlap in calendar months, season and period remain partly confounded and seasonality cannot be definitively ruled out. This study documents between-period differences in referral patterns and ED resource utilization; it does not establish causal effects of the pandemic or confirmed improvements in the appropriateness of care, particularly given the absence of validated acuity or severity measures and of institutional data on admission pathways. The observed changes may plausibly reflect enhanced inter-level coordination and a more selective referral process, but reduced access to care and organizational changes are equally compatible with the data. Future multi-center longitudinal studies incorporating interrupted time-series methodology, case-mix adjustment, and institutional-pathway data are needed to confirm these trends, clarify their mechanisms, and evaluate whether targeted interventions particularly regarding referral form completion and telemedicine integration can optimize the PC-to-ED interface.

## Figures and Tables

**Table 1:** Sociodemographic characteristics, referral origin by PC centre, and temporal distribution of referrals in the pre-pandemic (2019) and Later-Pandemic (2022) periods. PC centres are numbered in descending order of referral volume in 2019.

Variable	2019 (*n* = 946)	2022 (*n* = 1797)	*p*-Value
	*N* (%)	*N* (%)	
*Sex*			
Female	515 (54.4)	968 (53.9)	0.775
Male	431 (45.6)	829 (46.1)	
*PC centre of origin*			
PC Centre 1	255 (27.0)	428 (23.8)	<0.001
PC Centre 2	180 (19.0)	214 (11.9)	
PC Centre 3	159 (16.8)	278 (15.5)	
PC Centre 4	158 (16.7)	350 (19.5)	
PC Centre 5	71 (7.5)	96 (5.3)	
PC Centre 6	33 (3.5)	196 (10.9)	
PC Centre 7	23 (2.4)	107 (6.0)	
PC Centre 8	9 (1.0)	49 (2.7)	
Other centres	58 (6.1)	79 (4.4)	
*Day of the week*			
Monday	214 (22.6)	360 (20.0)	0.026
Tuesday	161 (17.0)	390 (21.7)	
Wednesday	223 (23.6)	401 (22.3)	
Thursday	168 (17.8)	340 (18.9)	
Friday	180 (19.0)	306 (17.0)	
*Work shift*			
Morning (08:00–15:00)	543 (57.4)	993 (55.3)	0.283
Afternoon (15:00–21:00)	403 (42.6)	804 (44.7)	

**Table 2:** Reason for referral and quality of referral form completion in the pre-pandemic (2019) and Later-Pandemic (2022) periods. Quality criteria assessed per Irazábal–Gutiérrez/Morera methodology.

Variable	2019 (*n* = 946)	2022 (*n* = 1797)	*p*-Value
	*N* (%)	*N* (%)	
*Reason for referral*			
Specialist assessment	878 (92.8)	1668 (92.9)	0.282
Request for complementary test	68 (7.0)	128 (7.1)	
Request for admission	1 (0.1)	0 (0.0)	
Appointment request	1 (0.1)	0 (0.0)	
*Referral form completeness*			
Clinical history present	894 (94.5)	1765 (98.2)	<0.001
Physical examination present	768 (81.2)	1504 (83.7)	0.097
Diagnostic impression present	423 (44.7)	899 (50.1)	0.008

**Table 3:** Distribution of referrals by diagnostic specialty in the pre-pandemic (2019) and Later-Pandemic (2022) periods (p<0.001 for overall between-period comparison).

Specialty	2019 (*n* = 946)	2022 (*n* = 1797)	*p*-Value
	*N* (%)	*N* (%)	
Gastroenterology	163 (17.2)	164 (9.1)	<0.001
Cardiology	147 (15.5)	248 (13.8)	
Pulmonology	122 (12.9)	257 (14.3)	
COVID-19	0 (—)	139 (7.7)	
Neurology	98 (10.4)	104 (5.8)	
Hematology	67 (7.1)	48 (2.7)	
ENT (Otorhinolaryngology)	61 (6.4)	75 (4.2)	
Orthopedics	55 (5.8)	330 (18.4)	
Infectious Diseases	54 (5.7)	9 (0.5)	
Urology	51 (5.4)	83 (4.6)	
Ophthalmology	28 (3.0)	109 (6.1)	
Dermatology	38 (4.0)	36 (2.0)	
Endocrinology	17 (1.8)	21 (1.2)	
Maxillofacial Surgery	15 (1.6)	0 (0.0)	
Nephrology	5 (0.5)	35 (1.1)	
Psychiatry	8 (0.8)	22 (1.2)	
General Surgery	7 (0.7)	62 (3.5)	
Gynecology	3 (0.3)	12 (0.7)	
Rheumatology	3 (0.3)	15 (0.8)	
Allergology	0 (0.0)	8 (0.4)	
Vascular Surgery	0 (0.0)	21 (1.1)	
Social Work	2 (0.2)	0 (0.0)	

**Table 4:** Emergency department length of stay, complementary investigations, treatments, and patient outcomes in the pre-pandemic (2019) and Later-Pandemic (2022) periods.

Variable	2019 (*n* = 946)	2022 (*n* = 1797)	*p*-Value
	*N* (%)	*N* (%)	
*ED length of stay*			
<3 h	642 (67.9)	588 (32.7)	<0.001
3–6 h	123 (13.0)	769 (42.8)	
6–12 h	18 (1.9)	195 (10.9)	
>12 h	163 (17.2)	245 (13.6)	
*Complementary investigations and treatments*			
Urgent blood tests	652 (68.9)	1011 (56.0)	<0.001
Plain radiography	539 (57.0)	1043 (58.0)	0.597
Advanced imaging (US or CT)	173 (18.3)	273 (15.2)	0.037
Urgent specialist consultation	172 (18.2)	384 (21.4)	0.048
IV treatment or plaster cast	419 (44.3)	573 (31.9)	<0.001
*Outcomes*			
ED observation area admission	120 (12.7)	157 (8.7)	<0.001
Hospital admission	647 (68.4)	196 (10.9)	<0.001
Referral to another centre	116 (12.3)	16 (0.9)	<0.001

US: ultrasound; CT: computed tomography; IV: intravenous; ED: emergency department.

**Table 5:** Multivariable logistic regression models adjusting for seasonal confounding in the two primary binary outcomes. Study period (post- vs. pre-pandemic), calendar season, age (continuous), and sex were entered simultaneously as covariates. Reference category for period: pre-pandemic (2019). Reference category for season: winter. Reference category for sex: female.

	Hospital Admission		Urgent Blood Tests	
Predictor	OR (95% CI)	*p*-Value	OR (95% CI)	*p*-Value
Period (post- vs. pre-pandemic)	0.062 (0.037–0.106)	<0.001	0.470 (0.326–0.678)	<0.001
Age (per year)	0.975 (0.969–0.980)	<0.001	0.979 (0.975–0.983)	<0.001
Season (overall)	NS ^†^		NS (p=0.166)	
Sex (male vs. female)	1.022 (0.830–1.259)	0.836	0.992 (0.847–1.161)	0.918
Model $\chi^{2}$	1094.2		142.2	
Global *p*-value	<0.001		<0.001	
Nagelkerke $R^{2}$	0.464		0.068	
Correct classification	82.1%		62.6%	

OR: odds ratio; CI: confidence interval; NS: not statistically significant (p≥0.05). ^†^ Season was not globally significant; a marginal association was observed for autumn (p=0.012), a season concentrated in the pre-pandemic observation window. No other category reached significance.

## Data Availability

The original contributions presented in this study are included in the article. Further inquiries can be directed to the corresponding author.

## Abbreviations

The following abbreviations are used in this manuscript:

COVID-19	Coronavirus Disease 2019
CT	Computed Tomography
ED	Emergency Department
ENT	Ear, Nose and Throat (Otorhinolaryngology)
EU	European Union
GP	General Practitioner
IV	Intravenous
NHS	National Health System
PC	Primary Care
SD	Standard Deviation
SPSS	Statistical Package for the Social Sciences
US	Ultrasound
WHO	World Health Organization
